# Cooperative Effects of FOXL2 with the Members of TGF-β Superfamily on FSH Receptor mRNA Expression and Granulosa Cell Proliferation from Hen Prehierarchical Follicles

**DOI:** 10.1371/journal.pone.0141062

**Published:** 2015-10-23

**Authors:** Ning Qin, Xian-Cong Fan, Xiao-Xing Xu, Thobela Louis Tyasi, Shi-Jun Li, Ying-Ying Zhang, Man-Li Wei, Ri-Fu Xu

**Affiliations:** 1 Department of Animal Genetics, Breeding and Reproduction, College of Animal Science and Technology, Jilin Agricultural University, Changchun, People’s Republic of China; 2 Department of Animal Genetics, Breeding and Reproduction, College of Animal Science and veterinary medicine, Huazhong Agricultural University, Wuhan, People’s Republic of China; China Agricultural University, CHINA

## Abstract

Forkhead box L2 (FOXL2) is a member of the forkhead nuclear factor 3 gene family and plays an essential role in ovarian growth and maturation in mammals. However, its potential effects and regulative mechanism in development of chicken ovarian prehierarchical follicles remain unexplored. In this study, the cooperative effects of FOXL2 with activin A, growth differentiation factor-9 (GDF9) and follistatin, three members of the transforming growth factor beta (TGF-β) superfamily that were previously suggested to exert a critical role in follicle development was investigated. We demonstrated herein, using *in-situ* hybridization, Northern blot and immunohistochemical analyses of oocytes and granulosa cells in various sizes of prehierarchical follicles that both *FOXL2* transcripts and FOXL2 proteins are predominantly expressed in a highly similar expression pattern to that of *GDF9* gene. In addition, the *FOXL2* transcript was found at lower levels in theca cells in the absence of *GDF9*. Furthermore, culture of granulosa cells (GCs) from the prehierarchical follicles (6–8 mm) in conditioned medium revealed that in the pcDNA3.0-FOXL2 transfected GCs, there was a more dramatic increase in *FSHR* mRNA expression after treatment with activin A (10 ng/ml) or GDF9 (100 ng/ml) for 24 h which caused a stimulatory effect on the GC proliferation. In contrast, a significant decrease of *FSHR* mRNA was detected after treatment with follistatin (50 ng/ml) and resulted in an inhibitory effect on the cell proliferation. The results of this suggested that FOXL2 plays a bidirectional modulating role involved in the intracellular *FSHR* transcription and GC proliferation via an autocrine regulatory mechanism in a positive or negative manner through cooperation with activin A and/or GDF9, and follistatin in the hen follicle development. This cooperative action may be mediated by the examined Smad signals and simultaneously implicated in modulation of the *StAR*, *CCND2*, and *CYP11A1* expression.

## Introduction

Development of hen ovarian follicles is a complicated and highly regulated process in which various endocrine, paracrine, and autocrine factors within the follicles act in a spatial and temporal manner to control and coordinate the growth and development of the oocyte, granulosa and theca cell layers [1–3]. Implications in this process are not only members of the glycoprotein hormone family of gonadotropins (such as follicle-stimulating hormone[FSH]), but also a wide variety of local intra-ovarian factors that play essential roles in regulating normal follicle development and oocyte maturation by mediating cellular and tissue level communication; these include transcription factors such as Forkhead box L2 protein (FOXL2) and members of the transforming growth factor beta (TGF-β) superfamily, including growth differentiation factor-9 (GDF9), follistatin and activin [4–6]. FOXL2 as a member of the winged helix/forkhead transcription factor family includes 39 known members in the human and mouse genomes, and manifests a variety of functions; such as acting as transcriptional activators and repressors [7]. It is a protein composed of 305 amino acids encoded by single-exon *FOXL2* gene in chicken [8]. The *FOXL2* gene was initially reported to express in less differentiated GCs of small and medium follicles in human, mouse and goat [9,10], and likely plays a significant role in granulosa cell differentiation, follicle development and maintenance[11,12]. Recent studies demonstrated that FOXL2 is involved in granulosa cell proliferation and folliculogenesis by co-regulating with mothers against decapentaplegic homolog2/3 (Smad2/3), the transcription of the *Fshb* gene that encodes the functional subunit β of FSH in mammals [13, 14]. Additionally, FOXL2 also exhibits a transcriptional repressor of steroidogenic acute regulatory protein (*StAR*), P450scc (*CYP11A*) and cyclin D2 (*CCND2*) genes, markers of ovarian follicle proliferation and differentiation [15, 16]. In the chicken, expression of *FOXL2* gene was primarily detected in developing follicles from the ovaries at embryonic day (E) 7, E14 of incubation and the adult ovary using qRT-PCR and Western blot analysis [8]. However, detailed spatiotemporal localizations of *FOXL2* transcript and FOXL2 protein, and its action in various follicles are poorly defined in hen.

In hen ovarian development, both follicular viability and associated differentiation following follicle selection are dependent on FSH stimulation and the expression of FSH receptor (FSHR) in granulosa cells [17]. It has been confirmed that comparatively high levels of *FSHR* mRNA are expressed in the granulosa layer from the individual prehierarchical follicles of 6–8 mm in diameter [17, 18]. In this process, the members of TGF-β superfamily, GDF9, activin A and follistatin have distinct functions in follicular development and growth by influence on the expression of *FSHR* gene in mammals and chicken [4–6, 19, 20]. Of which, the *GDF9* gene is specifically expressed in oocytes and essential for female fertility in chicken, human, sheep, and mice[3, 4, 19, 21]. GDF9 has been shown to control folliculogenesis by acting on GC in developing follicles [4], and to play a key role in promoting the growth, development and differentiation of cultured ovarian follicles [21, 22].

The activin A is composed of two beta A-subunits, β_A_ and β_A_, which was originally isolated from follicular fluid as a factor stimulating the FSH release from the pituitary [23], and exerts an autocrine and/or paracrine effect on ovarian follicle development [5, 20]. The chicken activin/inhibin β_A_ subunit gene (*INHBA*) has been cloned and showed that the *INHBA* gene mRNA was primarily expressed in the granulosa layer of the preovulatory follicles [20, 24]. The activin A can act to increase the granulosa cell number in folliculogenesis, but this effect could be inhibited by follistatin, an activin-binding protein that has been demonstrated to induce atresia of large antral follicles in mammals [5, 23]. Moreover, activin and GDF9 can induce the follistatin transcription in the primary GCs, but FOXL2 functions to negatively regulate GDF9 and activin-stimulated follistatin transcription in the human ovary [3, 25]. Treatment with activin A resulted in the increase of *FSHR* mRNA levels in cultured granulosa cells [17]. Activin A signals by complexing with its own membranes type I (TGFBR1) and type II (TGFBR2) serine/threonine kinase receptor and activating SMAD2/3 intracellular signaling [26]. Therefore, it was speculated that a cooperation of FOXL2 and the members of TGF-β superfamily (activin A, GDF9 and follistatin) may require for normal prehierarchical follicle development by controlling the expression of *FSHR* transcript and its biological effect in hen ovary. However, the localization and expression of activin A and follistatin in hen ovarian follicles are not known, and furthermore, the actions and mechanism of FOXL2 with the three members in follicle development remain poorly understood.

The objectives of this study were to characterize the detailed expression patterns and localizations of *FOXL2* mRNA and FOXL2 protein in prehierarchical follicles, and then to investigate potential roles for FOXL2 in regulating *FSHR* transcription and GC proliferation by the involvement of activin A, GDF9 and follistatin in hen follicle development.

## Materials and Methods

### Birds and sampling

Laying hens of Lohmann brown commercial line were reared in laying batteries according to the management reported by us [27]. All layers (n = 100) sampled for this experiment were randomly selected from the population and sacrificed at 21 weeks of age. Variously sized follicles were removed from the hen ovaries based on the method of Stepińska and Olszańska (1996) [28]. A representative portion of each ovary was taken and immediately frozen in liquid nitrogen, and stored at -80°C; and another equal part of the tissue was fixed using 4% neutral-buffered formalin at 4°C, and then transferred to 70% ethanol for subsequent embedding into paraffin wax. All procedures performed in animals were approved by the Institutional Animal Care and Use Committee of Jilin Agricultural University (Changchun, China).

### Quantitative real-time PCR

Total RNA was extracted from frozen ovarian tissues using TRIzol Reagent according to the manufacturer’s protocols (Gibco BRL, Grand Island, NY, USA). RNA quality and quantity were then assessed using a ND-1000 spectrophotometer (NanoDrop Technologies, Wilmington, DE, USA). The samples were treated with DNase (Promega, Madison, WI, USA) to remove genomic DNA contamination. Reverse transcription (RT) of RNA was performed in 30-μl reaction volumes using a First Strand Synthesis Kit (QIAGEN, Crawley, UK) based on the manufacturer’s protocol. Both RT-negative (containing template RNA but no reverse transcriptase enzyme) and RT water (containing reverse transcriptase but no template RNA) negative controls were used in every cDNA reaction. All samples were stored at -80°C until further use.

To assess mRNA expression of the candidate genes in GCs and follicular tissues using real-time quantitative reverse transcriptase PCR (qRT-PCR), specific sets of primer pairs were designed using the Primer Premier 5.0 program [29]. The primers used for *FOXL2* gene: forward 5’-CAACCTCAGCCTCAACGAGT-3’ and reverse 5’- GACATCTGGCAAGAGGCGTA -3’. The *18S rRNA* gene was used as an inner control in each reaction system: forward 5’-TTCCGATAACGAACGAGAC-3’ and reverse 5’-GACATCTAAGGGCATCACAG -3’. The other primers utilized for amplification of the *FSHR*, *INHBA*, *FST*, *GDF*9, *STAR*, *CCND2*, *CYP11A1* and *TGFBR1*genes were list in Table 1. All primer pairs were tested using both standard FastStart and SybrGreen PCR reagents and conditions. Examination of qRT-PCR was carried out in 25 μl containing primers at a final concentration of 150 nm each, with 5 μl of the RT reaction diluted 1/20, and qPCR Mastermix Plus for SYBRgreen I (Invitrogen, Carslbad, CA, USA) included according to the manufacturer’s instructions. All reactions were performed in triplicate using an ABI Prism 7200 Sequence Detection System (Applied Biosystems, Foster City, CA, USA). Formation of single products was confirmed by both gel electrophoresis and by examining the dissociation curve, and PCR products were confirmed by sequencing. Using the 2^-ΔΔCt^ method, mRNA expression results were normalized against *18S rRNA* as internal control.

**Table 1:** Primer pairs designed for quantitative real-time PCR.

Gene	Forward primer (5′ - 3′)	Reverse primer (5′ - 3′)	Accession No.	Size
*FOXL2*	CAACCTCAGCCTCAACGAGT	GACATCTGGCAAGAGGCGTA	NM_001012612.1	309 bp
*FSHR*	AATACCCTGCTAGGACTG	GAATACCCATTGGCTCA	NM_205079.1	238 bp
*INHBA*	AGGATGCCTTTGCTTTG	CTGGGTGATGTTAGGTCTG	U42377.1	231 bp
*FST*	ACAAGACCGACCTCAGCA	TTTCCCATCTAAGCCACA	NM_205200.1	275 bp
*GDF9*	ACTTTCACTCGGTGGATT	ATGCTGGGACATACTTGG	AY566700.2	175 bp
*STAR*	GCCAAAGACCATCATCAAC	TCCCTACTGTTAGCCCTGA	NM_204686.2	141bp
*CCND2*	AACTTGCTCTACGACGACC	TTCACAGACCTCCAACATC	NM_204213.1	150bp
*CYP11A1*	GCTTTGCCTTGGAGTCTGTG	TCGGTGCTCTTGCGTTGC	NM_001001756.1	227bp
*TGFBR1*	GCTGCGGACAACAAAGAC	ATGCCTAACTGCCAACCC	NM_204246.1	285bp
*18SrRNA*	TTCCGATAACGAACGAGAC	GACATCTAAGGGCATCACAG	AF173612.1	139 bp
*18SrRNA*	TCTTAGTTGGTGGAGCGATTT	CAAGCTGAGCCAGTCAGTGTAG	AF173612.1	208bp*

Note: The candidate gene *FOXL* encodes forkhead box L2 (FOXL2); *FSHR*, follicle-stimulating hormone receptor (FSHR); *INHBA*, activin/inhibin beta A-subunit; *FST*, follistatin (FST); *GDF9*, growth differentiation factor-9 precursor (GDF9); *STAR*, steroidogenic acute regulatory protein; *CCND2*, cyclin D2; *CYP11A1*, cytochrome P450, family 11, subfamily A, polypeptide 1; *TGFBR1*, transforming growth factor, beta receptor I (activin A receptor type II-like kinase); *18S rRNA*, 18S ribosomal RNA (18S rRNA). For the subsequent *in-situ* hybridization and Northern blot analysis, cRNA probes corresponding to the sense and antisense strands of PCR products of *FOXL2* and *GDF9* shown in the gene list in this Table were prepared in advance (*, the 208 bp fragment for 18S rRNA gene was used in Northern blot analysis).

### Histological processing and in-situ hybridization

Method of preparing paraffin sections of hen prehierarchical follicles and further histological processing were as our previously described [30]. After the sections were mounted on slides, a modified H&E staining procedure was used, as described by Zheng (2005) [31]. The sections were examined using a JNOEC XS-213 biological microscope (Jiangnan Optics & Electronics Co., Ltd. Nanjing, China) at magnifications of ×10, ×40 and ×200.

Frozen ovarian tissues were serially sectioned at a thickness of 10 μm on a cryostat for *in-situ* hybridization (ISH) experiments, using Digoxin (DIG)-labeled cRNA probes corresponding to the genes listed in Table 1, and their sense sequence probes against the genes were used to confirm the specificity of the binding. The DIG-labeled *in-situ* hybridization was performed as previously described with slight modifications [30]. mRNA expression of the candidate gene was then detected with a peroxidase-labeled anti-DIG antibody (dilution, 1:1000; Roche Diagnostics, Indianapolis, IN, USA) and chromogenically developed by incubating the slides with DAB staining solution (containing 3, 3’-diaminobenzidine 50 mg, 0.05 M Tris buffer (TB; 0.05 M Tris-hydrochloride buffer, pH 7.5) for 5 min at room temperature, and the sections were then counterstained with hematoxylin for morphological observation.

### Northern blot analysis and immunohistochemistry

The granulosa and theca cell layers were taken from follicles according to Rangel *et al* (2009) [32], washed in phosphate-buffered saline (pH 7.4, Qiagen), and stored at -80°C until later use. Abundances of mRNA expression were evaluated by Northern blot analysis using chicken *FOXL2* and *GDF9* cDNA probes as previously described [8]. The cRNA probes were prepared using the RT-PCR fragments (Table 1).The probes were confirmed by sequencing, and the probes were randomly labeled with [α-^32^P] dCTP using Rediprime (Invitrogen) and hybridized to the membrane with RapidHyb Buffer (Invitrogen) according to the manufacturer’s protocol.

Paraffin tissue sections were prepared for further immunohistochemical processing as described above and then iimmunohistochemical staining was performed as described [31], with some modifications to localize the candidate proteins in the follicles. Following deparaffinization, re-hydration and enzyme digestion, slides were incubated overnight using antibodies (in S1 Table) that were diluted with a mixture of 10% BS, 3% bovine serum albumin (BSA) and PBT at 4°C, and then incubated at room temperature for 1 h with secondary antibody labeled with horseradish peroxidase (HRP). After three washes with PBT for 4 min each, the sections were incubated for 30 min in 100 ml of DAB staining solution with 30% H_2_O_2_), and the stained sections were then counterstained in hematoxylin, cleared, and mounted with DPX (Leica Microsystems, Heidelberg, Germany). Photomicrographs of the sections were subsequently taken.

### Culture of granulosa cells and cell transfection for FOXL2 expression

Culture of granulosa cells from hen prehierarchical follicles was performed according to the published method [19]. Construction of recombinant plasmid vector and transfection for *FOXL2* gene was performed as described previously [13]. Briefly, the granulosa cells were grouped randomly were transfected by a reconstructed plasmid pcDNA3.0-FOXL2 (our unpublished data) and pcDNA3.0 blank vector (Invitrogen, Carlsbad, CA, USA) using Lipofectamine 2000 transfection reagent (Invitrogen, USA). Cultures were conducted in the presence or absence of activin A (10 ng/ml), GDF9 (100 ng/ml) or FST (50 ng/ml) to the basal medium as aforementioned. After 24 h of the culture, cells were collected for immunoblot analysis and RNA extracted for qRT-PCR analysis.

### Transfection of siRNA

Specific siRNAs targeting *FOXL2* gene were designed using an Invitrogen siRNA Wizard v3.1 [33]. All designed siRNA sequences were blasted against the chicken genome database to eliminate cross-silence phenomenon with nontarget genes. The nucleotide sequences were as follows: siRNA seq-1, 5′- GUCCGGGAUCUACCAGUACATT -3′; and siRNA seq-2, 5′- GGAUCUACCAGUACAUCAUCATT -3′. Scrambled siRNA that does not target any gene was used as the negative control siRNA: 5′- GUAUGACGAUCCGACCUAGUCTT -3′. As mentioned above, GCs were plated in 24-well plates and the siRNAs were transfected into the culture cells with Lipofectamine 2000 (Invitrogen, USA) according to the manufacturer's instructions.

### Cell proliferation assays

Following the procedure of cell transfection for FOXL2 expression, a modified [^3^H]-thymidine incorporation into GCs method was performed, as described by Hutchison (2007)[34]. In this determination, [^3^H]-thymidine (1.5 μCi/ml) was added to each plate in the presence or absence of activin A (10 ng/ml), GDF9 (100 ng/ml), FST (50 ng/ml), or FSH (50 ng/ml) as aforementioned. After 24 h, the GCs were rinsed twice with PBS to remove the excess [^3^H]-thymidine, and were then fixed with ice-cold trichloroacetic acid (50%) and lysed by NaOH (0.5 N). Five independent replicate experiments were carried out. The incorporated radioactivity in the cells was measured after resuspension by a scintillation counter.

### Immunoblot analysis

Following cell transfection test, western blot analysis for FOXL2, phospho-Smad1, 2 and3 and total Smad1, 2 and 3 protein was conducted as previously described [35] using total cellular extracts. Briefly, equivalent amounts of protein were separated by 10% (w/v) SDS- polyacrylamide gel under reducing conditions and electro-transferred to Protran nitrocellulose membranes (Whatman, Dassel, Germany). The affinity purified antibody for FOXL2 (Santa Cruz, CA, USA) and (phospho-) Smad1 (Rockford, IL, USA), Smad 2 and3 (Invitrogen, Carlsbad, CA, USA) was used (S2 Table). The horseradish peroxidase-conjugated anti-rabbit or anti-mouse IgG secondary antibody was incubated for 2 h at room temperature. Blots were subsequently performed with ECL western blotting agent (Rockford, IL, USA) for 5 min and exposed to X-ray film for 1–5 min. The outcome was visualized by the ECL Plus Western blotting detection system according to the manufacturer's instructions. Anti-β-actin (dilution 1:1000, Boster, China) antibody acted as loading control.

### Data analysis

Statistical calculation was conducted using the SPSS12.0 software package [36]. All the experiments were repeated at least three times using different batches of sampled birds. In the experiment entailing mRNA expression levels by qRT-PCR analysis, four amplified products from independent reactions per individual were quantified. After confirmation of normal distributions for parametric analysis, the data were analyzed using a one-way ANOVA with Tukey’s multiple-comparison test when more than two groups were involved, or using a Student’s *t* test when treatment and control groups were compared. *P <* 0.01 or *P <* 0.05 was accepted to be statistically significant.

## Results

### Localization and expression of *FOXL2* mRNA in ovarian follicles

**Fig 1:**
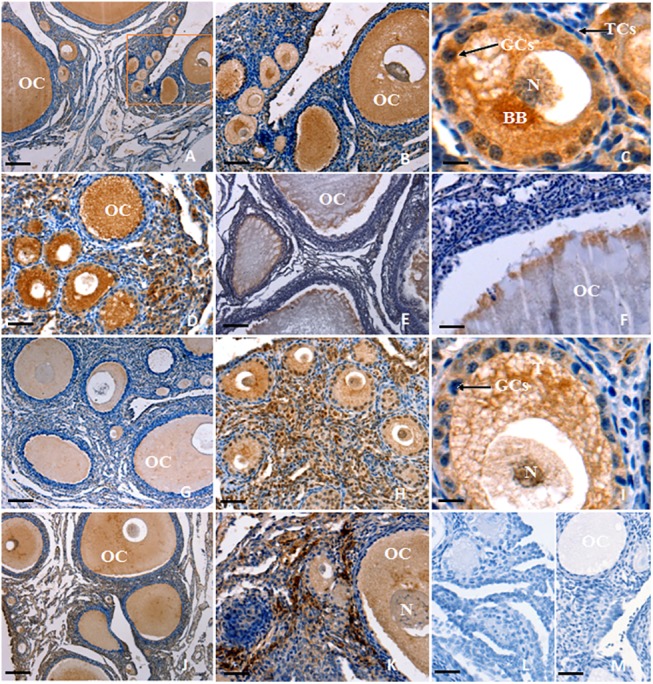
Localization of *FOXL2* and *GDF9* transcripts in hen ovarian follicles. *In-situ* hybridization was performed to localize *FOXL2* (A-F) and *GDF9* transcripts (G-K) in ovarian follicle sections using *FOXL2* or *GDF9* gene anti-sense probes. Panel A shows strong signal for *FOXL2* mRNA expression in all oocytes (OC) and granulosa cells (GCs) within variously sized follicles (×10); Panels B-D show localization of *FOXL2* mRNA in the OC and GCs of primary and early prehierarchichal follicles (PF) (< 300 μm in diameter; B, ×40; D, ×200). The *rectangular area* in panel A indicates the location of panel B. Panels E-F, localization of *FOXL2* mRNA in the PF (600 μm to 8 mm in diameter; E, ×10; F, ×40). Panel G, localization of *GDF9* transcripts in the differently sized follicles (×10); Panels H-I, OC, and GC within primary and early (20–200 μm diameter; H, ×40; I, ×200) PF; Panels J-K, OC, and GC within the more developed (400 μm to 8 mm diameter; J,×10; K,×40) PF; Panel L and M, one of the negative control section for *FOXL2* and *GDF9*, respectively. The ovarian tissue sections were hybridized with *FOXL2* or *GDF9* sense probes. Five birds were used for *in-situ* hybridization, six serial sections per biopsy were examined and representative microscopic fields were selected. Theca cells (TCs), somatic cells (SCs), Balbiani body (BB), and nucleus (N). Scale bar = 100 μm.

**Fig 2:**
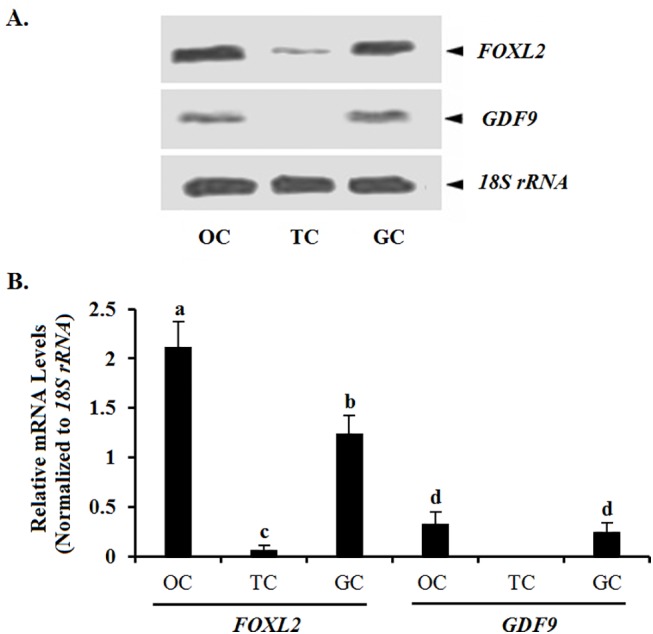
Northern blot analysis of the expression of *FOXL2* and *GDF9* mRNA in oocytes (OC), theca cells (TCs), and granulosa cells (GCs) from prehierarchical follicles (6–8 mm in diameter). Relative expression was analyzed by Northern blot using *FOXL2* and *GDF9* cRNA probes; an *18S rRNA* cRNA probe (208 bp) was used as a loading control. The results were examined on a 1% agarose gel containing 1 M formaldehyde as assessed by electrophoresis. Hybridization signal intensity was quantified densitometrically after phosphorimaging (shown in A), and normalized for loading by comparison to the hybridization signal for *18S rRNA*. The signal intensity of *FOXL2* or *GDF9* genes was expressed as the ratio*18S rRNA* signal in arbitrary units shown in B (n = 5 per mean ± SEM). Statistical significance was set at *P <* 0.01, and marked with different superscript letters a, b, c, and d.

This study initially localized *FOXL2* transcripts in variously sized prehierarchical follicles (PF) using ISH. It was indicated that *FOXL2* mRNA is predominantly expressed in the oocytes and undifferentiated GCs from primary follicles (30–90 μm in diameter) and PF of 60 μm to 8 mm in diameter (Fig 1A–1F). By contrast, a very low level of *FOXL2* transcript was found in the theca cell (TC) layer (Fig 2). Similarly, the transcripts for *GDF9* gene was also detected to localize in the oocytes and GCs within the every follicle examined (Fig 1G–1K). However, the positive signal of *GDF9* mRNA expression was absent or undetectable in the TCs (Fig 2).

**Fig 3:**
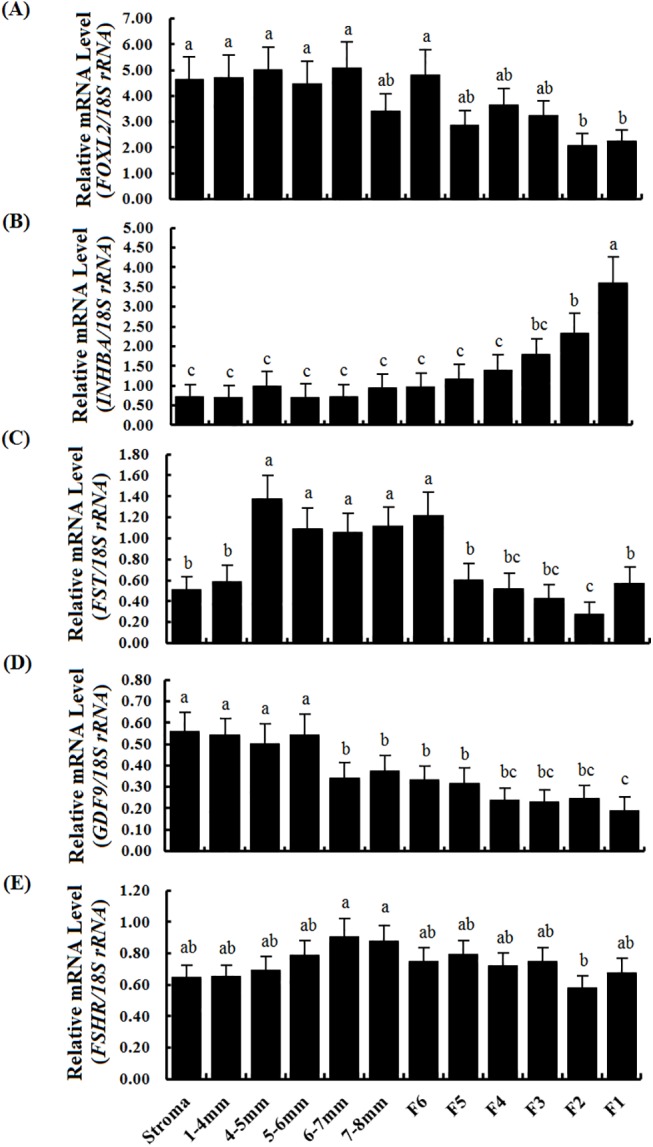
Quantification of mRNA expression levels of the *FOXL2* gene in variously sized follicles form freshly collected ovaries. The data are the mean ± SEM from 10 hens (n = 10), and bars with different letters above them differ significantly in the amount of mRNA expression as normalized to the *18S rRNA* gene (*P* < 0.05). The ovarian stroma contains matrix tissue from large follicles (≥1 mm in diameter), small follicles (<1 mm in diameter), atretic follicles, somatic and other cells. 1–4 mm, the follicles were from 1 to 4 mm in diameter; 4–5 mm, >4 mm but ≤5 mm in diameter; 5–6 mm, >5 mm but ≤ 6 mm in diameter; and the others followed similarly. (A) Quantification of *FOXL2* mRNA levels by RT-qPCR. (B) Levels of *INHBA* mRNA. (C) Levels of *FST* mRNA. (D) Levels of *GDF9* mRNA. (E) Levels of *FSHR* mRNA. Horizontal ordinate represents the examined follicle sizes as indicated in (E).

As follicle development, *FOXL2* mRNA was maintained at a high level in the prehierarchical follicles (1–8 mm diameter), ovarian stroma and the larger preovulatory (F6-F1) follicles (Fig 3A). For comparison, transcript abundance for *GDF9*, *INHBA*, *FST* and *FSHR* genes was also quantified by qRT-PCR. Of which, the average mRNA level for the *FOXL2* gene remained at the highest level from the PF of 1-4mm in diameter to the largest F1 (≥40 mm size) follicles(Fig 3A). Expression levels of *INHBA* gradually increased with stage of development and dramatically reached a highest level in preovulatory F2-F1 follicles (Fig 3B). Levels of *FST* mRNA were highest in mid-sized (4–8 mm diameter) and preovulatory (F6, in diameter 9–12 mm) follicles (Fig 3C), whereas the average mRNA expression level of the *GDF9* gene was the lowest than that of *FOXL2*, *INHBA*, or *FST*. Interestingly, the *FSHR* mRNA was expressed stably from the1-4 mm PF to the largest preovulatory F1 (Fig 3E).

### Expression pattern of FOXL2 protein in prehierarchical follicles

**Fig 4:**
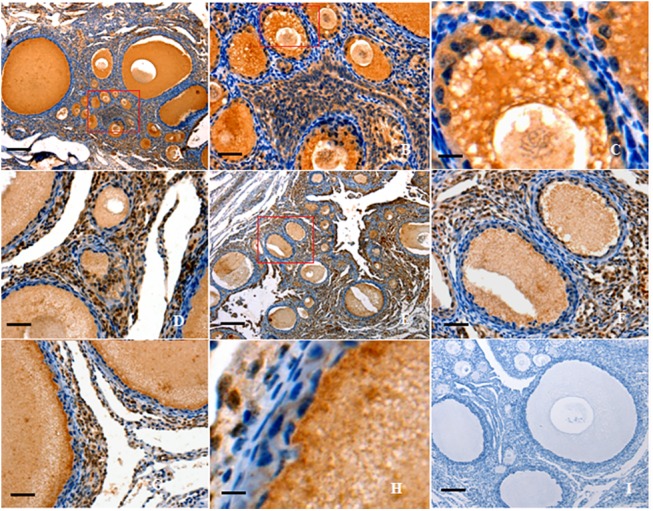
Immunohistochemical analysis of FOXL2 and GDF9 protein expression in the ovarian follicle. Paraformaldehyde-fixed tissue sections were immunostained using anti-chicken FOXL2 (A-D) or anti-chicken GDF9 (E-H) as described above. Panel A, strong staining is observed in all oocytes (OC) and granulosa cells (GCs) within the variously sized follicles (×10). Panel B, the amplified primary follicles and prehierarchical follicles (×40), paralleling the one marked in the box of panel A; Panel C, the amplified small-sized PF follicles with two or three layers of GC(×200), paralleling the one marked in the box of panel B. Panel D, the amplified large PF follicles with more layers of GCs (×40). Panel E, a distinct positive staining was seen in the all follicles observed (×10). Panel F, in parallel to the one marked in the box of panel E, strong staining was observed in OC and GCs (×40). Panel G, the amplified prehierarchical follicle (×40); Panel H, the amplified large PF follicles with more layers of GCs (×200). Panel I, one of the negative control for FOXL2, the sections were immunostained with pre-immune serum, no significant expression was detected (similar negative control for GDF9 is not given here). Negative control sections were prepared with the primary antibody pre-incubated with a blocking peptide for 1 h at room temperature and for 2 h at 4°C. Five birds were used for immunohistochemical analysis and representative microscopic fields were selected. Scale bar = 100 μm.

**Fig 5:**
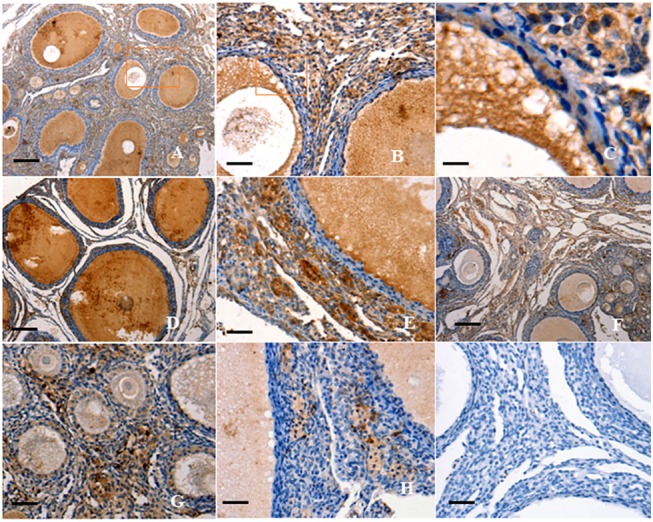
Immunohistochemical analysis of follistatin and activin A protein expression in the ovarian follicle. The sections were immunostained using anti-chicken follistatin (A-E) or anti-chicken activin A (F-H). Panel A shows strong staining of follistatin expression in OC and GCs within the variously sized follicles (×10). Panels B, localization of follistatin protein in the OC and GCs of primary and early prehierarchichal follicles (×40) (< 300 μm in diameter), paralleling the one marked in the box of panel A; Panels C, paralleling the one marked in the box of panel B (×200); Panels D-E, localization of follistatin in large PF (600 μm to 8 mm in diameter; D, ×10; E, ×40); Panel F, staining of activin A expression was observed in OC and GCs within variously sized follicles (×10); Panel G, localization of activin A protein in the OC, GCs, and TCs of primary and early prehierarchichal follicles (< 300 μm in diameter; ×40); Panel H, localization of activin A in large PF (1 to 8 mm in diameter; ×40); Panel I, negative control for activin A, with no significant expression detected (similar negative control for follistatin is not given here). Scale bar = 100 μm.

FOXL2 and GDF9 were predominantly expressed in oocytes and GCs within PF at the every developmental stage (Fig 4). The expression pattern was fairly similar for FOXL2 and GDF9. As follicle growth, the positive immunostaining was observed in the oocytes and GCs of primary (Fig 4B and 4F), small prehierarchical (60–300 μm in diameter) follicles (Fig 4C and 4G) and the larger (1–8 mm in diameter) follicles (Fig 4A and 4E). A detectable staining for FOXL2 protein was found in TC layers within the follicles examined. A strongly positive staining for follistatin was also observed in the oocytes and GCs within the various sized follicles (Fig 5A–5E). The spatiotemporal expression of follistatin shared a highly similar pattern to that of FOXL2 or GDF9 expression. Whereby, activin A was not only expressed in oocytes and GCs, but also in TCs of primary and early PF (< 300 μm in diameter) (Fig 5F and 5G).

### Effect of FOXL2 on expression of *FSHR* mRNA and proliferation of granulosa cells in vitro

**Fig 6:**
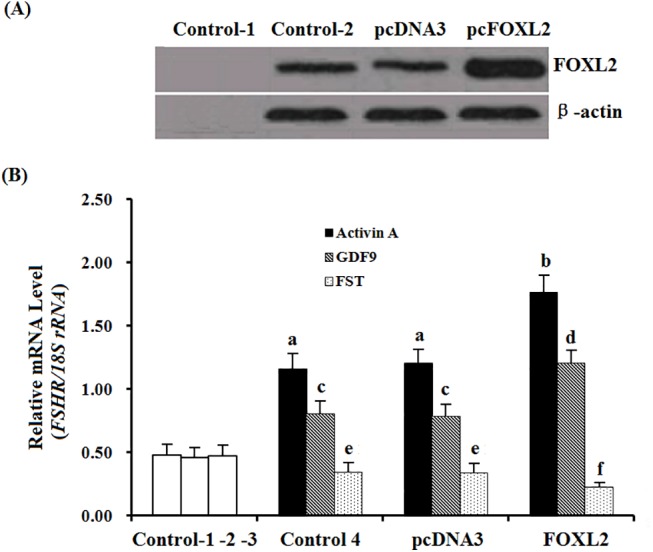
Variation of the expression of *FSHR* mRNA in the granulosa cells transfected by reconstructed pcDNA3.0-FOXL2 plasmids and cultured with activin A, GDF9 and follistatin. (A), the total cellular extracts were subjected to Western blot analysis. Chicken β-actin (42 kDa) was used as the loading control. Control 1, negative control, without cellular extracts; Control 2, the granulosa cells cultured with the basal medium, no transfection with pcDNA3.0 blank vector or the reconstructed plasmid pcDNA3.0-FOXL2 vector, indicating the endogenous FOXL2 was expressed. pcDNA3, the cells were transfected only by pcDNA3.0 blank vector, also indicating the endogenous FOXL2. pcFOXL2, the cells were transfected by the reconstructed plasmid pcDNA3.0-FOXL2 vector, indicating a transient expression of a recombinant FOXL2 protein. (B), Variation of the expression of *FSHR* mRNA in the cells responding to the expression of FOXL2, cultured in presence or absence of activin A, GDF9 or FST to the basal medium as indicated. Control 1, the granulosa cells cultured with the basal medium, no transfection with pcDNA3.0 blank vector or the reconstructed plasmid pcDNA3.0-FOXL2 vector, indicating the endogenous *FSHR* mRNA was expressed. Control 2, the cells were transfected only by pcDNA3.0 blank vector, also indicating the endogenous *FSHR* mRNA expression. Control 3, expression of *FSHR* mRNA in the cells transfected by the pcDNA3.0-FOXL2 plasmid. Control 1–3, absent of any of the activin A, GDF9 and follistatin, Control 4, the cells cultured with the same basal medium to control 1–3, but present of the activin A(10 ng/ml), GDF9 (100 ng/ml) and follistatin (100 ng/ml) as indicated. pcDNA3.0, the cells were transfected by pcDNA3.0 blank vector and cultured same to the control 4; FOXL2, the cells were transfected by the reconstructed plasmid pcDNA3.0-FOXL2 vector. Each treatment was performed in triplicate at 38.5°C in 5% CO_2_ for 24 h. The values on the bar graphs are the mean ± SEM of 10 hens (n = 10) form a representative experiment. For each treatment, bars with different letter superscripts above them are significantly different in expression levels of *FSHR* mRNA in the cultured granulosa cells (*P <* 0.05).

As shown in Fig 6, a high expression of FOXL2 was detected in the undifferentiated GCs from the prehierarchal follicles (6–8 mm in diameter) after transfected with the reconstructed pcDNA3.0-FOXL2, and endogenous expression of FOXL2 were also detectable by Western blotting in the cells with and without pcDNA3.0 vector transfected (Fig 6A). Under the stimulation of overexpression of FOXL2 factor alone, no significant increase or decrease of *FSHR* mRNA was observed in the pcDNA3.0-FOXL2 vector-transfected cells. In the treatment with activin A (10 ng/ml) or GDF9 (100 ng/ml), there was a notable increase of *FSHR* mRNA in the cultured cells for 24 h (Fig 6B); in contrast, *FSHR* mRNA was decreased after cultured with FST (100 ng/ml) compared to the control. Surprisingly, there was a more dramatic increase of *FSHR* mRNA expression in the pcDNA3.0-FOXL2 transfected cells under the treatment with activin A or GDF9 for 24 h, in contrast, a more notable decrease of *FSHR* mRNA was detected after treatment with FST. By comparison, silencing *FOXL2* expression, the Effect of *FOXL2* on the expression of *FSHR* mRNA was significantly suppressed in the cultured GCs with activin A, GDF9 and FST, respectively (S1 Fig and S2 Fig).

**Fig 7:**
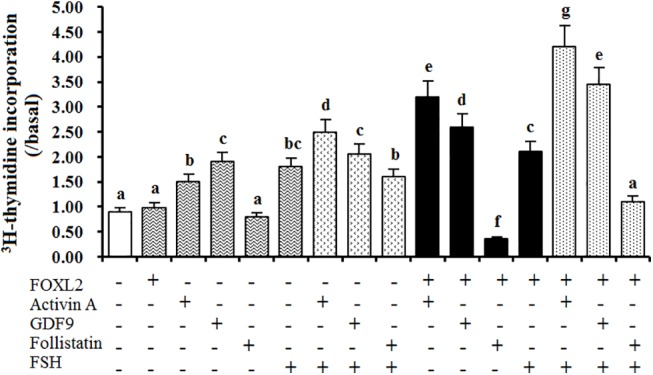
Effects of FOXL2 and the three factors on proliferation rate of cultured hen GCs. Thymidine incorporation was determined in GCs from prehierarchichal follicles (6 to 8 mm in diameter) transfected with or without the reconstructed pcDNA3.0-FOXL2 cultured for 24 h in the presence or absence of activin A (10 ng/ml), GDF9 (100 ng/ml), FST (50 ng/ml), or FSH (50 ng/ml) as list in the figure. Results are expressed as means ± SEM in relation to values in the absence of treatment (basal state). Different letters above the bars indicate that difference was significant (P<0.05). The Five independent experiments were carried out in triplicate. The results are representative of at least three independent experiments.

In Fig 7, activin A, GDF9 and FSH had significant effect on the cell proliferation (P < 0.05), respectively. No significant effect of FOXL2 or FST alone was observed (P > 0.05). Among the combined treatment groups, treatment of FOXL2 with activin A, (activin A+FSH), and (GDF9+FSH) combination had the greatest stimulatory effect on the cell proliferation (P < 0.05), respectively. Significant effect was observed the group of activin A and FSH, or FOXL2 and GDF9 treatment (P < 0.05) also. The combination of FST and FSH, GDF9 and FSH, or FOXL2 and FSH had a similar effect on the cell proliferation to that of the FSH treatment, but the treatment with FOXL2 and FST lead to a significant decrease in [^3^H]-thymidine incorporation level(P < 0.05). Knock-downing *FOXL2* expression, the Effect of *FOXL2* on the cell proliferation was significantly attenuated under the treatment with activin A, GDF9 and FST (S3 Fig).

### Induced Smad1, 2 and 3 phosphorylation and expression of *StAR*, *CCND2*, *CYP11A1* and *TGFBR1* mRNA by the TGF-β members in GCs from the prehierarchichal follicles

**Fig 8:**
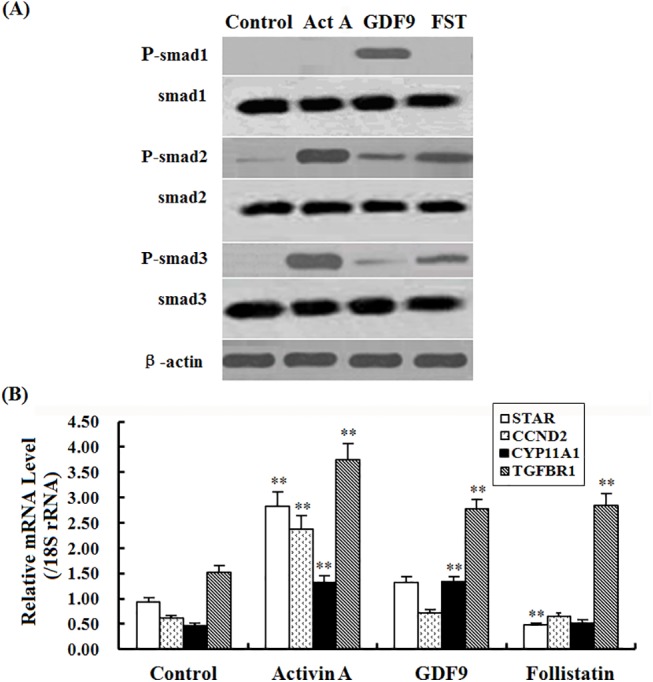
Phosphorylation of Smad1, Smad 2, and Smad 3 proteins and the expression of *StAR*, *CCND2*, *CYP11A1* and *TGFBR1* mRNA examined. Panel A, Phosphorylation of Smad1, 2, 3 proteins induced by activin A (10 ng/ml), GDF9 (100 ng/ml) or FST (50 ng/ml) in granulosa cells from the 6 to 8 mm prehierarchichal follicles. The β-actin was used as the loading control. Panel B, Expression of *StAR*, *CCND2*, *CYP11A1* and *TGFBR1* mRNA simulated by the TGF-β members for 24 h in the cultured GCs from prehierarchichal follicles (6 to 8 mm in diameter).The superscript symbol indicates that difference was significant ** P<0.01,* P<0.05.

GDF9 (100 ng/ml) was able to promote phosphorylation of Smad1 (mothers against decapentaplegichomolog1) in the GCs from the prehierarchal follicles (6–8 mm in diameter), but activin A (10 ng/ml) and FST (50 ng/ml) had no phosphorylation effect on Smad1 protein in the experimental condition (Fig 8A). activin A, GDF9 and FST was also able to induce phosphorylation of Smad2 and Smad3 in the GCs. Furthermore, activin A resulted in a significant elevation in mRNA expression levels of *StAR*, *CCND2*, *CYP11A1* and *TGFBR1* genes (P < 0.01; Fig 8B). GDF9 was only to promote the expression of *CYP11A1* and *TGFBR1* mRNA (P < 0.05). However, FST was able to significantly stimulate the decrease of *StAR* mRNA expression level, while it simultaneously led to a sharp increase in mRNA expression of *TGFBR1* gene (P < 0.01).

## Discussion

In this study, we initially localized the *FOXL2* transcript by using ISH in an attempt to explore the possible role of the *FOXL2* gene in the regulation of the prehierarchical follicular development in laying hens. The specifically positive signals for *FOXL2* transcripts found in oocytes and GCs from primary follicles and undifferentiated PF 60 μm to 8 mm in diameter suggests its functional importance in oocyte development and in GC proliferation and differentiation in the follicles. This spatiotemporal expression of the *FOXL2* transcript presented a very similar pattern to that of *GDF9* gene in the various sized follicles sampled, but this was not the case for TCs. Both transcripts localized in the GC layers as well as in the oocytes from the follicles were reinforced by Northern blot analysis. Previous report has demonstrated *GDF9* mRNA is expressed in GCs and oocytes of the 6- and 8- mm follicles of the hen by real-time RT-PCR [19]. Interestingly, changes of *FOXL2* mRNA expression levels were also revealed in a similar pattern to that of *GDF9* mRNA in the various staged follicles revealed using qRT-PCR, but the expression abundance between *FOXL2* and *GDF9* genes were divergent significantly (Fig 3). Some relevant studies indicated a critical role for the oocyte and granulosa cell derived GDF9 in normal follicular development [4, 21], particularly in stimulating granulosa cell proliferation as has been demonstrated in mammals [19]. The current data insinuated that FOXL2 factor may function temporally and spatially correlated with that of GDF9 with their different concentration dependent characteristics in the development of prehierarchical follicles, as recently reported for murine, goat, and human [3,14], but it need to be further confirmed at the protein level.

FOXL2 protein is required for granulosa cell differentiation and ovary maintenance in mammals [11, 15]. This study also showed the immunocytochemical localization of both FOXL2 and GDF9 proteins was highly similar to the patterns of *FOXL2* and *GDF9* transcripts detected by ISH. This result strongly suggested that the FOXL2 factor plays an indispensable, intra-ovarian local regulating role in oocyte development and GC proliferation and/or differentiation of the PF in a coordinated manner with GDF9 protein in hen. In the process of the ovary growth, many members of the TGF-β superfamily, including activin A and follistatin have also been demonstrated to exert an essential effect on GC proliferation and follicle development in rat, mouse, sheep and goat [37–40]. In this work, the predominant localization in oocytes and GC layers for both activin A and follistatin shared a highly similar pattern to that of FOXL2 or GDF9 expression by immunohistochemistry, but changes in mRNA expression levels were examined in an distinctly divergent manner between each other by using real-time RT-PCR, in accordance with previous studies in mammals [5, 41]. Although activin A and follistatin have previously been demonstrated expressed in gonadotropes of human and rat pituitary [42,43], this result indicated that the effects of activin A and follistatin on the follicle development were fulfilled by a local paracrine and/or autocrine regulation in hen ovary, as reported functions in mammals [5, 41, 44]. However, the intra-ovarian local role for FOXL2 and regulative mechanism cooperated with activin A, GDF9 and follistatin in hen follicle development are not known.

Prior to follicle selection, a higher level of *FSHR* mRNA in GCs may be required for the selection of PF 6–8 mm in diameter into the preovulatory hierarchy [17]. Herein, this study showed that a relatively high and stable expression abundance of *FSHR* mRNA was found in the various sized follicles (Fig 3). Consistent with the previously reported in chicken and mammals [19, 40], a stimulatory effect of activin A or GDF9 on the expression abundance of *FSHR* mRNA was found in the hen GCs *in vitro*. Moreover, as overexpression of FOXL2, a sharp increase of *FSHR* mRNA was detected under the treatment with activin A or GDF9 (Fig 6). This result primarily indicated that the stimulatory effect of activin A or GDF9 on expression of *FSHR* mRNA in the GC layers of ovarian follicle can be strongly elevated by action with FOXL2 factor. According to the previous reports, activin was originally identified in a positive feedback loop, secreted from the gonads to induce FSH secretion and FSHβ gene expression in pituitary gonadotrope [45]. Activin A plays an important role in the regulation of ovarian follicle development in goat, mouse and rat [38, 46, 47]; and GDF9 exert a critical function in granulosa cell and theca cell growth, as well as in the differentiation and maturation of the oocyte [4, 21, 22, 48]. Herein, our data demonstrated that the cooperative action of FOXL2 with activin A or GDF9 was able to significantly promote the cultured GC proliferation, in contrast, the stimulatory effect on the cell proliferation under the combination of FOXL2 with activin A and FSH, or GDF9 and FSH was more significant than that of the treatment with the combined FSH and activin A, FSH and GDF9, or FSH alone. So, this stimulatory effect on the cell proliferation was supposed mainly associated with the markedly elevated *FSHR* mRNA transcription level affected by the FOXL2 combination and the subsequent increase of FSH-responsiveness (to efficiently bind to FSHR) induced by activin A or GDF9 signaling. However, the action of activin A in this regulative process may depend on a different molecular mechanism to that of GDF9 protein. Attractively, our current results demonstrate that both activin A and GDF9 were able to induce phosphorylation of Smad2 and Smad3 in the GCs (Fig 8A). As shown in mammals [49, 50], activin A and GDF9 may activate the Smad 2/3 signal pathway in chicken GCs, respectively. This result seems contradictory with the previous studies on ovarian preovulatory follicles, which showed that the activin A signaling induces Smad2 activity, but not Smad3 in chicken granulosa cells [51]. But, the present data is concordant with previous studies showing that Smad2 and Smad3 are expressed mainly in undifferentiated or poorly differentiated granulosa cells (GCs) in rat [52], and both are expressed at specific stages during ovarian development [53]. Moreover, it is also possible that there are different receptor-regulated Smads signals between the developing prehierarchichal and preovulatory follicles. However, Smad2 and Smad3 were significantly expressed in cultured GCs in vitro [53]. Furthermore, GDF9 may also activate the Smad 1/5/8 signaling simultaneously, because GDF9 were also able to induce phosphorylation of Smad1 as well as Smad2 and Smad3 in the GCs (Fig 8A). So, we suggested that chicken GDF9 protein may play a promoting role, as showed in mammals [48, 50, 54, 55], in the GC proliferation of the prehierarchical follicles through two signalling systems: TGFβ/activin-smad pathway [49, 56] and GDF9/ BMPRII pathway [54, 57]. As described above, a generic mechanism of the Smads-mediated signal transduction was previously demonstrated [13, 26, 51, 56, 57]. Herein, a potential regulation pathway of FOXL2 involved in the hen follicle development that was coordinated by the members of TGF-β superfamily (including activin A and GDF9) was illustrated for further analysis of the effects of FOXL2 on *FSHR* mRNA expression (S4A Fig).

There have been many reports of direct interaction between FOXL2 and the Smad proteins, as intracellular signaling molecules transferring the signal induced by activin A or other members of TGF-β superfamily at the cell membrane to the nucleus [3, 12, 13, 25]. However, the cooperative effects for the intracellular FOXL2 and activin A or GDF9 on *FSHR* mRNA expression and GC proliferation from the prehierarchical follicles may be directly involved in by the SMADs proteins through a currently unknown mechanism (S4B Fig). Additionally, in the following studies, we found that the expression levels of the *StAR*, *CCND2*, *CYP11A1* and *TGFBR1* mRNA were simultaneously augmented in the GCs in corresponding to the phosphorylation of Smad 2/3 induced by activin A. Unexpectedly, the increase of mRNA expression levels were only observed in *CYP11A1* and *TGFBR1* genes in responding to activation of Smad2/3 and Smad1 under the treatment with GDF9. However, it was previously demonstrated FOXL2, a central transcription factor of ovary, represses key genes in granulosa cell differentiation including aromatase, P450scc, and cyclin D2 in human and mouse [15, 16].

It is known that FST was able to significantly inhibit the growth of the cultured isolated primary follicles by counteracting the effect of activin A in goats [38]. The current data demonstrated that FST was able to notably suppress the expression of *FSHR* mRNA in GCs by the involvement of FOXL2, and caused an inhibitory effect on the cell proliferation. In the adult ovary, pituitary FSH via interaction with its receptor (FSHR) is required for follicular development and GC proliferation and differentiation [58, 59]. Therefore, the down-regulation of *FSHR* mRNA expression in GCs was inferred to be associated with an inhibitory effect on follicular development by reducing the capability of FSH-responsiveness in the ovary; moreover, FOXL2 factor should be required for FST attenuating the *FSHR* mRNA expression. The consequences to suppress *FSHR* expression in GC layers have been shown to directly inhibit FSH action in rat [60]. This may be the main reason why no significant decrease of the [^3^H]-thymidine incorporation level was observed under the treatment of a combination of FST and FSH, or FOXL2 and FSH. Interestingly, our further examination found that Smad2/3 signaling was induced at this stage of the prehierarchical follicle development by treatment with FST (as by activin A), it was supported by the result of phosphorylation of Smad2 and 3 in the cultured GCs (Fig 8A). Moreover, the phosphorylation of Smads may directly lead to the dramatic increase of *TGFBR1* mRNA and the marked decrease of *STAR* mRNA expression in GCs after treatment with FST *in vitro* (Fig 8). However, the precise molecular mechanism of coordinative actions of FOXL2 and FST for repressing hen follicular development remains to be further explored (see S4 Fig).

## Conclusion

This study confirmed that FOXL2 plays a bidirectional modulating role involved in the intracellular *FSHR* transcription and granulosa cell proliferation via an autocrine regulatory mechanism in a positive or negative manner in cooperation with activin A and/or GDF9, and follistatin in the hen follicle development. The effects on the examined gene expression predicted a complicated mechanism of the FOXL2 actions cooperated with activin A, GDF9 or follistatin on *FSHR* mRNA expression and granulosa cell proliferation, and which was mediated by Smad proteins and simultaneously implicated in modulation of the *StAR*, *CCND2*, and *CYP11A1* expression.

## Supporting Information

S1 Fig Chicken *FOXL2* specific-siRNAs suppressed the *FOXL2* mRNA expression in the granulosa cells in vitro. (A), Expression of *FOXL2* mRNA was analyzed by real-time PCR. (B), Expression of FOXL2 protein was analyzed by Western blot analysis. β-actin (42 kDa) was used as the loading control. Data are presented as mean ± SEM from at least four independent experiments. Bars with superscript symbols (**) are significantly different (P<0.01).
(TIF)

S2 Fig Effect of knockdown *FOXL2* expression on the FSH receptor mRNA expression in the granulosa cells in vitro. Expression of *FSHR* mRNA was analyzed by real-time PCR in the cultured granulosa cells with or without activin A (10 ng/ml), GDF9 (100 ng/ml) and follistatin(50 ng/ml). Data are presented as mean ± SEM from at least four independent experiments. Bars with different superscript letters are significantly different (P<0.01). Blank control, the granulosa cells cultured with the basal medium, no transfection with the siRNAs and absent of any of the activin A, GDF9 and follistatin, indicating the endogenous *FSHR* mRNA was expressed. Negative control, the cells cultured with the same basal medium to the blank control, no transfection with the siRNAs, but present of the activin A, GDF9 and follistatin.
(TIF)

S3 Fig Effect of knockdown *FOXL2* expression on the proliferation of granulosa cells in vitro. Thymidine incorporation was determined in the GCs from prehierarchichal follicles (6 to 8 mm in diameter) transfected with or without the *FOXL2* specific-siRNAs cultured for 24 h in the presence or absence of activin A (10 ng/ml), GDF9 (100 ng/ml), FST (50 ng/ml), or FSH (50 ng/ml) as list in the figure. Results are expressed as means ± SEM in relation to values in the absence of treatment (basal state). Different letters above the bars indicate that difference was significant (P<0.05). The Five independent experiments were carried out in triplicate. The results are representative of at least three independent experiments.
(TIF)

S4 Fig Schematic illustration of the potential regulation pathway of FOXL2 involved in the hen follicle development that was coordinated by the members of TGF-β superfamily. (A), Regulation of FOXL2 coordinated with the exogenous or paracrinely released activin A, GDF9 and follistatin in FSH mRNA expression and GC proliferation in the cultured GCs. (B), Pathway of FOXL2 cooperated with the endogenous or autocrinely released activin A, GDF9 and follistatin in the GCs. But it is unclear how the FOXL2 interacted with the intracellular transcription factor Smads in the regulation of *FSHR* transcription, and how the autocrinely released members of TGF-β superfamily activated the Smads and then partnered with FOXL2 to regulate *FSHR* transcription. The molecular mechanism of chicken FOXL2 action still requires further confirmation and refinement.
(TIF)

S1 Table Antibodies and blocking peptides used for immunohistochememistry. (DOCX)

S2 Table Antibodies used for Western blot analysis. (DOCX)
