# Supplementary figures and images for: Cooperative Effects of FOXL2 with the Members of TGF-β Superfamily on FSH Receptor mRNA Expression and Granulosa Cell Proliferation from Hen Prehierarchical Follicles
Source: PLoS One. 2015 Oct 23;10(10):e0141062. doi: 10.1371/journal.pone.0141062 (PMC4619702; doi:10.1371/journal.pone.0141062)

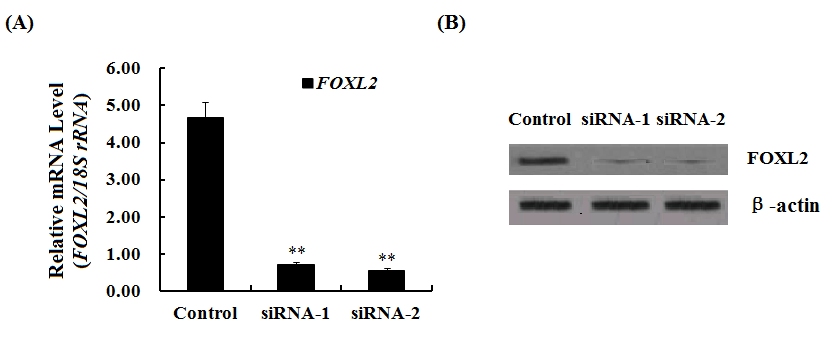

Supplement: S1 Fig — (A), Expression of FOXL2 mRNA was analyzed by real-time PCR. (B), Expression of FOXL2 protein was analyzed by Western blot analysis. β-actin (42 kDa) was used as the loading control. Data are presented as mean ± SEM from at least four independent experiments. Bars with superscript symbols (**) are significantly different (P<0.01). (TIF) [file pone.0141062.s001.tif]

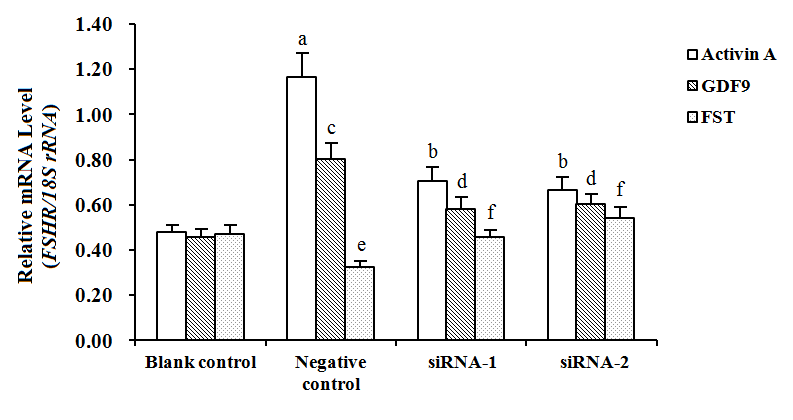

Supplement: S2 Fig — Expression of FSHR mRNA was analyzed by real-time PCR in the cultured granulosa cells with or without activin A (10 ng/ml), GDF9 (100 ng/ml) and follistatin(50 ng/ml). Data are presented as mean ± SEM from at least four independent experiments. Bars with different superscript letters are significantly different (P<0.01). Blank control, the granulosa cells cultured with the basal medium, no transfection with the siRNAs and absent of any of the activin A, GDF9 and follistatin, indicating the endogenous FSHR mRNA was expressed. Negative control, the cells cultured with the same basal medium to the blank control, no transfection with the siRNAs, but present of the activin A, GDF9 and follistatin. (TIF) [file pone.0141062.s002.tif]

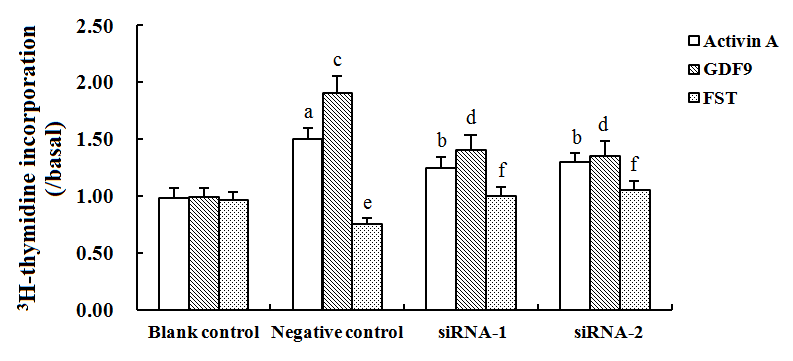

Supplement: S3 Fig — Thymidine incorporation was determined in the GCs from prehierarchichal follicles (6 to 8 mm in diameter) transfected with or without the FOXL2 specific-siRNAs cultured for 24 h in the presence or absence of activin A (10 ng/ml), GDF9 (100 ng/ml), FST (50 ng/ml), or FSH (50 ng/ml) as list in the figure. Results are expressed as means ± SEM in relation to values in the absence of treatment (basal state). Different letters above the bars indicate that difference was significant (P<0.05). The Five independent experiments were carried out in triplicate. The results are representative of at least three independent experiments. (TIF) [file pone.0141062.s003.tif]

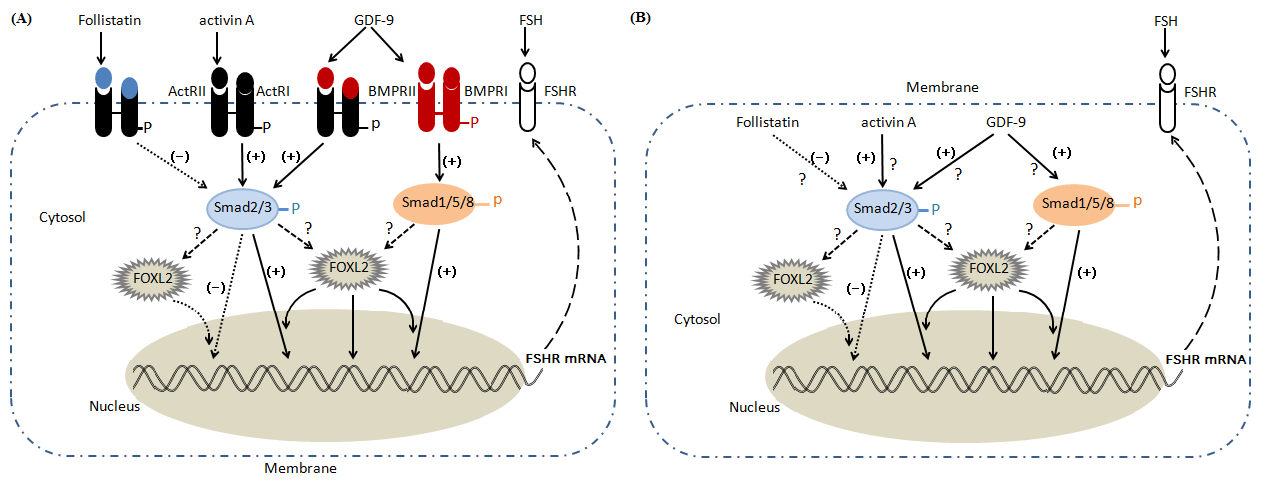

Supplement: S4 Fig — (A), Regulation of FOXL2 coordinated with the exogenous or paracrinely released activin A, GDF9 and follistatin in FSH mRNA expression and GC proliferation in the cultured GCs. (B), Pathway of FOXL2 cooperated with the endogenous or autocrinely released activin A, GDF9 and follistatin in the GCs. But it is unclear how the FOXL2 interacted with the intracellular transcription factor Smads in the regulation of FSHR transcription, and how the autocrinely released members of TGF-β superfamily activated the Smads and then partnered with FOXL2 to regulate FSHR transcription. The molecular mechanism of chicken FOXL2 action still requires further confirmation and refinement. (TIF) [file pone.0141062.s004.tif]
