# Supplementary material for: Cooperative Effects of FOXL2 with the Members of TGF-β Superfamily on FSH Receptor mRNA Expression and Granulosa Cell Proliferation from Hen Prehierarchical Follicles
Source: PLoS One. 2015 Oct 23;10(10):e0141062. doi: 10.1371/journal.pone.0141062 (PMC4619702; doi:10.1371/journal.pone.0141062)
Supplement: S1 Table — (DOCX) [file pone.0141062.s005.docx]

**S1 Table. Antibodies and blocking peptides used for immunohistochememistry**

| **Protein target** | **Primary**  **antibody** | **Dilution used** | **Antibody type** | **Secondary**  **antibody** | **Dilution used** | **Antibody type** | **Blocking peptide** |
| --- | --- | --- | --- | --- | --- | --- | --- |
| FOXL2 | Rabbit anti- FOXL2 | 1/1000 | Monoclonal | Goat anti-rabbit | 1/2000 | Monoclonal | FOXL2 internal region |
| activin A | Mouse anti- activin A | 1/1000 | Monoclonal | Rabbit anti-mouse | 1/2000 | Monoclonal | activin A residues 300-450 |
| GDF9 | Rabbit anti- GDF9 | 1/500 | Monoclonal | Goat anti-rabbit | 1/1000 | Monoclonal | GDF9 internal region |
| follistatin | Mouse anti- follistatin | 1/1000 | Monoclonal | Rabbit anti-mouse | 1/2000 | Monoclonal | follistatin residues 300-400 |

Note: Source of the antibodies against the chicken protein/peptide target list in this table: FOXL2 and GDF9 antibodies (Sigma, St Louis, MO, USA), activin A and follistatin (Santa Cruz, CA, USA). Antibody concentration: 1μg/ul, size: 100 ul.
