# Supplementary material for: Cooperative Effects of FOXL2 with the Members of TGF-β Superfamily on FSH Receptor mRNA Expression and Granulosa Cell Proliferation from Hen Prehierarchical Follicles
Source: PLoS One. 2015 Oct 23;10(10):e0141062. doi: 10.1371/journal.pone.0141062 (PMC4619702; doi:10.1371/journal.pone.0141062)
Supplement: S2 Table — (DOCX) [file pone.0141062.s006.docx]

**S2 Table. Antibodies used for Western blot analysis**

| **Protein target** | **Primary**  **antibody** | **Dilution used** | **Antibody type** | **Secondary**  **antibody** | **Dilution used** | **Originated** |
| --- | --- | --- | --- | --- | --- | --- |
| FOXL2 | Rabbit anti- FOXL2 | 1/2000 | Monoclonal | anti-rabbit IgG | 1/5000 | Santa Cruz, CA, USA |
| Smad1 | Rabbit anti- Smad1 | 1/5000 | Monoclonal | anti-rabbit IgG | 1/10000 | Rockford, IL, USA |
| Smad2 | Mouse anti- Smad2 | 1/6000 | polyclonal | anti-mouse IgG | 1/10000 | Invitrogen, Carlsbad, CA, USA |
| Smad3 | Mouse anti- Smad3 | 1/6000 | polyclonal | anti-mouse IgG | 1/10000 | Invitrogen, Carlsbad, CA, USA |
| pSmad1 | Rabbit anti- pSmad1 | 1/1000 | Monoclonal | anti-rabbit IgG | 1/3000 | Rockford, IL, USA |
| pSmad2 | Mouse anti- pSmad2 | 1/1000 | polyclonal | anti-mouse IgG | 1/6000 | Invitrogen, Carlsbad, CA, USA |
| pSmad3 | Mouse anti- pSmad3 | 1/1000 | polyclonal | anti-mouse IgG | 1/6000 | Invitrogen, Carlsbad, CA, USA |

Note: Source of the antibodies against the chicken protein/peptide target list in this table. pSmad : phospho-Smad. Antibody concentration: 1μg/ul, size: 100 ul.
